# Development and Validation of a Nomogram for Predicting Bronchopulmonary Dysplasia in Very-Low-Birth-Weight Infants

**DOI:** 10.3389/fped.2021.648828

**Published:** 2021-03-19

**Authors:** Jingdi Zhang, Chenghan Luo, Mengyuan Lei, Zanyang Shi, Xinru Cheng, Lili Wang, Min Shen, Yixia Zhang, Min Zhao, Li Wang, Shanshan Zhang, Fengxia Mao, Ju Zhang, Qianya Xu, Suge Han, Qian Zhang

**Affiliations:** ^1^Neonatal Intensive Care Unit, The First Affiliated Hospital of Zhengzhou University, Zhengzhou, China; ^2^Orthopedics Department, The First Affiliated Hospital of Zhengzhou University, Zhengzhou, China; ^3^Health Care Department, The First Affiliated Hospital of Zhengzhou University, Zhengzhou, China; ^4^Children Health Care Department, Children's Hospital Affiliated of Zhengzhou University, Zhengzhou, China; ^5^Medical Record Management Department, The First Affiliated Hospital of Zhengzhou University, Zhengzhou, China

**Keywords:** NT-proBNP level, bronchopulmonary dysplasia, LASSO regression, nomogram, very-low-birth-weight infants

## Abstract

**Background:** Bronchopulmonary dysplasia is a common pulmonary disease in newborns and is one of the main causes of death. The aim of this study was to build a new simple-to-use nomogram to screen high-risk populations.

**Methods:** In this single-center retrospective study performed from January 2017 to December 2020, we reviewed data on very-low-birth-weight infants whose gestational ages were below 32 weeks. LASSO regression was used to select variables for the risk model. Then, we used multivariable logistic regression to build the prediction model incorporating these selected features. Discrimination was assessed by the C-index, and and calibration of the model was assessed by and calibration curve and the Hosmer-Lemeshow test.

**Results:** The LASSO regression identified gestational age, duration of ventilation and serum NT-proBNP in the 1st week as significant predictors of BPD. The nomogram-illustrated model showed good discrimination and calibration. The C-index was 0.853 (95% CI: 0.851–0.854) in the training set and 0.855 (95% CI: 0.77–0.94) in the validation set. The calibration curve and Hosmer-Lemeshow test results showed good calibration between the predictions of the nomogram and the actual observations.

**Conclusion:** We demonstrated a simple-to-use nomogram for predicting BPD in the early stage. It may help clinicians recognize high-risk populations.

## Introduction

In 1967, Northway identified a new disease in preterm infants with hyaline membrane disease, which is currently known as neonatal respiratory distress syndrome (RDS), and named the new disease bronchopulmonary dysplasia (BPD) (1). The rate of BPD ranges from 10.2 to 24.8% among different European countries (2). As the gestational age (GA) and birth weight (BW) of prematurity have decreased, the incidence of BPD has risen. With the evolution of perinatal medical and respiratory support technology, the mortality of extremely preterm infants has been dramatically reduced, but the incidence of BPD has not changed over the last decades due to the increased survival of preterm newborns (3). BPD is still the major cause of mortality among preterm babies in Level III neonatal units in China (4). Some longitudinal studies have shown that BPD patients develop respiratory infections more frequently in the first 2 years of life and that their airway function is reduced during childhood (5). BPD also affects neurological development. Guo et al. found that BPD had a significant association with cerebral palsy (CP), highlighting the fact that BPD is a risk factor for CP (6). At the practical level, this knowledge requires us to pay more attention to the quality of life and long-term outcomes of these infants. Thus, measures for the prevention and prediction of BPD are urgently needed.

The least absolute shrinkage and selection operator method (LASSO) is a powerful machine learning method to select the most relevant risk factors. It adds a penalty term for the shrinkage of the parameter estimates to the least-squares loss function. To eliminate variables, the L1 penalty could force coefficient estimates to zero (7). This method could circumvent the necessity for explicit multiple testing correction and prevent overfitting of the model (8). It is increasingly common in the genetic field and others, but it is seldom used in BPD prediction (9). The first prediction model was proposed by Cohen A in 1983 and included two variables, namely, the fraction of inspired O2 (FiO2) duration and the intermittent mandatory ventilation (IMV) time (10). More candidate risk factors were taken into account in later research, and most studies selected either the amount of oxygen administered or the positive inspiratory pressure in their model. M. Laughon et al. (11) reported a prediction model that was developed as a web-based calculator that included parameters such as GA, BW, sex, FiO_2_, ethnicity and respiratory support to estimate the probability of death and severity of BPD. However, regarding ethnicity, the study included only white, black and Hispanic subjects, and it was not adapted to Asian populations. In addition to the predictors above, some biological markers were found to be useful in identifying BPD early. There is evidence showing an association between increased N-terminal-pro brain natriuretic peptide (NT-proBNP) levels and neonatal morbidities such as BPD and pulmonary hypertension (12), but it has not been included in a model establishment currently. Therefore, our aim of the current study was to develop a clinical prediction model, including clinical characteristics and biochemical indexes, to identify very-low-birth-weight infants with high BPD risk.

## Materials and Methods

### Data Source

Patients were recruited from The First Affiliated Hospital of Zhengzhou University Neonatal Intensive Care Unit (NICU) Department II from January 2017 to December 2020. This data was collected from medical records. Patients with GAs <32 weeks, with BWs below 1,500 g and who were admitted to the NICU within 24 h after birth were included, and the length of stay was more than 1 week. The exclusion criteria were patients with inherited disease, metabolic disorders and multiple malformations. Our primary outcome was BPD/death. In accordance with the NICHD definitions, we evaluated whether patients were treated with oxygen >21% for at least 28 days, and the time point of assessment was 36 weeks postnatal menstrual age (PMA) or at discharge to the home, whichever came first (13). The study involving human participants was reviewed and approved by the ethics committee of the First Affiliated Hospital of Zhengzhou University (Ethical code: 2019-KY-95).

### Risk Factors

Based on clinical experience and prior literature, we collected data containing maternal diseases and neonatal clinical features, maternal diseases including intrauterine growth restriction (IUGR), antenatal steroid use, meconium staining, gestational hypertension, gestation diabetes mellitus (GDM), premature rupture of membranes (PROM), mothers' age, intrauterine distress, and neonatal characteristic including GA, BW, sex, delivery route, plasma NT-proBNP level on day 1, 3, and 7, RDS, neonatal asphyxia, apnea, multifunctional organ failure (MODS), disseminated intravascular coagulation (DIC), respiratory failure (RF), heart failure (HF), sepsis, pneumonia, patent ductus arteriosus (PDA), pulmonary hemorrhage, the usage of pulmonary surfactant (PS) and the total MV time within the 1st week. The diagnosis was assessed during the perinatal period. All risk factors were measured independently. The NT-proBNP data in this study are expressed as the logarithm of the NT-proBNP level based one.

The administration of antenatal corticosteroid was defined as dexamethasone 6 mg intramuscular injection (4 times every 12 h) and finished within 7 days before delivery.

Diagnosis of IUGR: IUGR is an abnormal situation in which a fetus cannot reach its genetic growth potential due to pathological processes. IUGR was classified as early IUGR and late IUGR at the 32nd gestational week. Infants born before the 32nd gestational week were defined as IUGR by any of the following conditions: (1) fetal abdominal circumference (AC)/estimated fetal weight (EFW) < the 3rd percentile or umbilical artery-absent end-diastolic flow (UA-AEDF) or (2) AC/EFW < the 10th percentile combined with a uterine artery-pulsatility index (UtA-PI) > the 95th percentile and/or a UA-PI> 95th percentile. All parameters were measured by Doppler ultrasound (14).

Diagnosis of RDS: RDS is a common disease among premature newborns. It was diagnosed mainly by clinical characteristics, including progressive dyspnea, and X-ray findings presented with reduced transmittance.

Diagnosis of HF: HF was defined clinically by the presence of clinical features. It mainly contained tachycardia (heart rates were over 150~160 times per minute when they were quiet), Traube's bruit, cardiomegaly, blood pressure decreased or normal, cyanosis, tachypnea (respiratory rate more than 60 times per minute), rhonchi or moist rales and hepatomegaly.

Diagnosis of RF: RF was defined by clinical features and laboratory results. It was defined as the presence of three depression signs: moaning, cyanosis, intractable apnea and tachypnea (respiratory rate more than 60 times per minute). It also included arterial partial pressures of O2 (PaO2), arterial partial pressures of CO2 (PaCO2) and pH values.

Diagnosis of meconium staining: Meconium staining of the amniotic fluid was defined clinically as the presence of meconium at any point during labor and delivery by the obstetrical team and recorded in the medical record (15).

Diagnosis of intrauterine fetal distress (16): Fetal distress was defined clinically as the presence of prolonged decelerations, repetitive moderate to severe variable decelerations or repetitive late decelerations not responsive to intrauterine resuscitative measures.

Diagnosis of gestational hypertension (17): Blood pressure criteria for hypertension in pregnancy were based on AHA/ACC definitions. It was defined as a systolic blood pressure (SBP) of 140 mmHg or more, a diastolic blood pressure (DBP) of 90 mmHg or more, or both after 20 weeks of gestation.

Diagnosis of gestational diabetes mellitus GDM (18): GDM was defined by the 50 g-OGTT test.

Diagnosis of PROM (19): PROM was defined as the rupture of the amniotic membranes with release of the amniotic fluid more than 1 h before the onset of labor.

Diagnosis of apnea of prematurity (20): Apnea of prematurity was defined as 20 s of no breathing or 10 s of no breathing concurrent with either a heart rate <80 beats per minute or oxygen desaturation >85%.

The usage of PS (21): If it was deemed necessary, we administered pulmonary surfactant (PS) as early as possible in the course of RDS. Babies with RDS were given PS when FiO_2_ requirements >0.3 in preterm infants ≤ 26 weeks gestation, and the others were given when FiO_2_ requirements >0.4. We used the INSURE (intubate-surfactant-extubate to CPAP) technique to administer PS, and the dose of poractant was 200 mg/kg.

### Statistical Analysis

Baseline maternal and perinatal characteristics were compared between premature infants with BPD and those without BPD. Continuous variables were tested by *t* test and presented as X± standard deviation if they were normally distributed. If not, they were tested by the Mann-Whitney *U* test and presented as medians with quartile ranges. Categorical data were analyzed by the χ^2^ test as numbers (n) and percentages (%). A two-sided *P* value of <0.05 was used as the criterion to indicate a statistically significant difference.

### Establishment and Validation of the Predictive Model and Nomogram

We divided premature infants admitted to the hospital before May 2019 into a training set, and the rest were included in the validation set. LASSO regression was used to select the optimal predictive factors. The minimum turning parameter λ was determined with cross-validation. Then, we used multivariable logistic regression to build the prediction model. The nomogram was constructed based on the methods described previously, and it was subjected to 1,000 bootstrap resamples for internal validation. We calculated the C-index to assess the discrimination performance. A calibration curve was plotted to quantify the calibration ability, combined with the Hosmer-Lemeshow test. An insignificant Hosmer-Lemeshow test statistic implied good calibration. Then, the prediction model was built in the training set, and all potential predictors were applied. The performance of the model in terms of discrimination and calibration was assessed by the C-index and calibration curve, respectively, in the validation group. All statistical analyses were performed using R software (Version 3.6.3, https://www.R-project.org).

## Results

### Patient Characteristics

From January 2017 to December 2020, 499 preterm infants whose gestational age was below 32 weeks and birth weight was under 1,500 g were admitted to the First Affiliated Hospital of Zhengzhou University. Five patients were diagnosed with congenital diseases and metabolic disorders, and 23 preterm babies died within the first 7 days. In addition, 36 patients were excluded because of incomplete medical records. A total of 435 preterm infants were included in this study, and 20 infants died before the BPD criteria could be evaluated. The causes of death included sepsis (*n* = 3); pulmonary hemorrhage (*n* = 2); necrotizing enterocolitis (*n* = 6); circulatory failure (*n* = 2); respiratory failure (*n* = 6); and DIC (*n* = 1). We divided 362 infants into a training set and 73 into a validation set according to the admission data (May 2019). In the training set, 195 were diagnosed with BPD, and 19 died. There were significant differences in delivery, GA, BW, meconium staining, gestational hypertension, neonatal asphyxia, HF, RF, pneumonia, pulmonary hemorrhage, the usage of PS, the total MV time and the NT-proBNP level on days 3 and 7 (*P* < 0.05). The demographic characteristics of the training and validation groups are summarized in Table 1.

**Table 1:** Clinical features in patients.

	**Training set (*****n*** **=** **362)**			**Validating set (*****n*** **=** **73)**		
	**No BPD group**	**BPD or death group**	***P*-value**	**No BPD group**	**BPD or death group**	***P*-value**
	**(*n* = 148)**	**(*n* = 214)**		**(*n* = 38)**	**(*n* = 35)**	
**Maternal disease**						
Meconium staining (%)	22 (14.9)	55 (25.7)	0.019	11 (28.9)	11 (31.4)	1
Gestational hypertension (%)	81 (54.7)	93 (43.5)	0.045	12 (31.6)	13 (37.1)	0.8
GDM (%)	18 (12.2)	31 (14.5)	0.632	8 (21.1)	6 (17.1)	0.899
PROM (%)	44 (29.7)	52 (24.3)	0.303	11 (28.9)	9 (25.7)	0.963
Mothers' age (year; median, IQR)	29 (27, 35)	31 (28, 34)	0.459	30 (28, 32)	31 (27, 35)	0.249
Intrauterine distress (%)	26 (17.6)	47 (22.0)	0.373	7 (18.4)	9 (25.7)	0.639
IUGR (%)	8 (5.4)	15 (7.0)	0.692	2 (5.3)	5 (14.3)	0.363
Antenatal steroids (%)	53 (35.8)	57 (26.6)	0.086	10 (26.3)	8 (22.9)	0.944
**Neonatal features**						
Male (%)	71 (48.0)	120 (56.1)	0.158	16 (42.1)	20 (57.1)	0.294
Caesarean delivery (%)	125 (84.5)	159 (74.3)	0.029	29 (76.3)	22 (62.9)	0.319
GA (week, median (IQR))	30.57 (29.71, 31.29)	29.00 (28.18, 29.71)	<0.001	30.43 (29.46, 30.96)	29.14 (27.71, 29.71)	<0.001
<28 weeks (%)	2 (1.4)	36 (16.8)		2 (5.3)	12 (34.3)	
28 ≤ GA <30 weeks (%)	45 (30.4)	132 (61.7)		13 (34.2)	15 (42.9)	
30 ≤ GA <32 weeks (%)	101 (68.2)	46 (21.4)		23 (60.5)	8 (22.9)	
BW (g, median (IQR))	1,250 (1,100, 1,385)	1,140 (1,000, 1,250)	<0.001	1,290 (1,150, 1,350)	1,050 (910, 1,200	<0.001
1 min Apgar score (median (IQR))	8.00 (7.00, 9.00)	7.00 (5.00, 8.00)	<0.001	8.00 (7.00, 9.00)	8.00 (6.00, 9.00)	0.203
5 min Apgar score (median (IQR))	9.00 (8.00, 10.00)	8.00 (7.00, 9.00)	<0.001	9.00 (9.00, 10.00)	9.00 (8.00, 9.00)	0.013
NT pro-BNP at the 1st day (ng/L, median, IQR)	8.05 (7.36, 8.91)	8.28 (7.65, 8.98)	0.065	8.27 (7.57, 9.39)	8.48 (7.80, 9.19)	0.795
NT pro-BNP at the 3rd day (ng/L, median, IQR)	9.07 (8.25, 9.97)	9.81 (8.62, 10.46)	<0.001	8.36 (7.78, 9.75)	9.99 (8.86, 10.46)	0.001
NT pro-BNP at the 7th day (ng/L, median, IQR)	7.58 (7.01, 7.95)	8.30 (7.52, 9.29)	<0.001	7.14 (6.60, 7.81)	7.71 (7.01, 8.96)	0.016
MV time (day; median, IQR)	0.00 (0.00, 0.00)	2.00 (0.00, 7.00)	<0.001	0.00 (0.00, 0.00)	3.00 (0.00, 7.00)	<0.001
Neonatal asphyxia (%)	42 (28.4)	101 (47.2)	<0.001	13 (34.2)	17 (48.6)	0.314
RDS (%)	140 (94.6)	202 (94.4)	1	35 (92.1)	35 (100.0)	0.268
Apnea (%)	16 (10.8)	27 (12.6)	0.721	0 (0.0)	5 (14.3)	0.051
MODS (%)	0(0.0)	6 (2.8)	0.102	0 (0.0)	2 (5.7)	0.437
DIC (%)	5 (3.4)	17 (7.9)	0.118	0 (0.0)	1 (2.9)	0.967
Heart failure (%)	32 (21.6)	84 (39.3)	0.001	3 (7.9)	12 (34.3)	0.012
Respiratory failure (%)	31 (20.9)	95 (44.4)	<0.001	9 (23.7)	16 (45.7)	0.083
PDA (%)	51 (31.1)	65 (35.5)	0.449	5(13.2)	17 (48.6)	0.002
Pneumonia (%)	13 (8.8)	36 (16.8)	0.041	9 (23.7)	10 (28.6)	0.835
Sepsis (%)	7 (4.7)	11 (5.1)	0.755	0 (0.0)	5 (14.3)	0.051
Pneumorrhagia (%)	2 (1.4)	17 (7.9)	0.012	1 (2.6)	3 (8.6)	0.549
PS (%)			0.001			0.001
0	28 (18.9)	15 (7.0)		17 (44.7)	4 (11.4)	
1	120 (81.1)	196 (91.6)		20 (52.6)	23 (65.7)	
2	0 (0.0)	3 (1.4)		1 (2.6)	8 (22.9)	

*IQR, Interquartile range; GDM, gestation diabetes mellitus; PROM, premature rupture of membranes; IUGR, intrauterine growth restriction; GA, gestational age; BW, birth weight; MV, mechanical ventilation; RDS, neonatal respiratory distress syndrome; MODS, multifunctional organ failure; DIC, disseminated intravascular coagulation; PDA, patent ductus arteriosus. PS, pulmonary surfactant. All NT pro-BNP data were present as the logarithm of NT-proBNP level based one*.

### Development of Prediction Model

**Figure 1:**
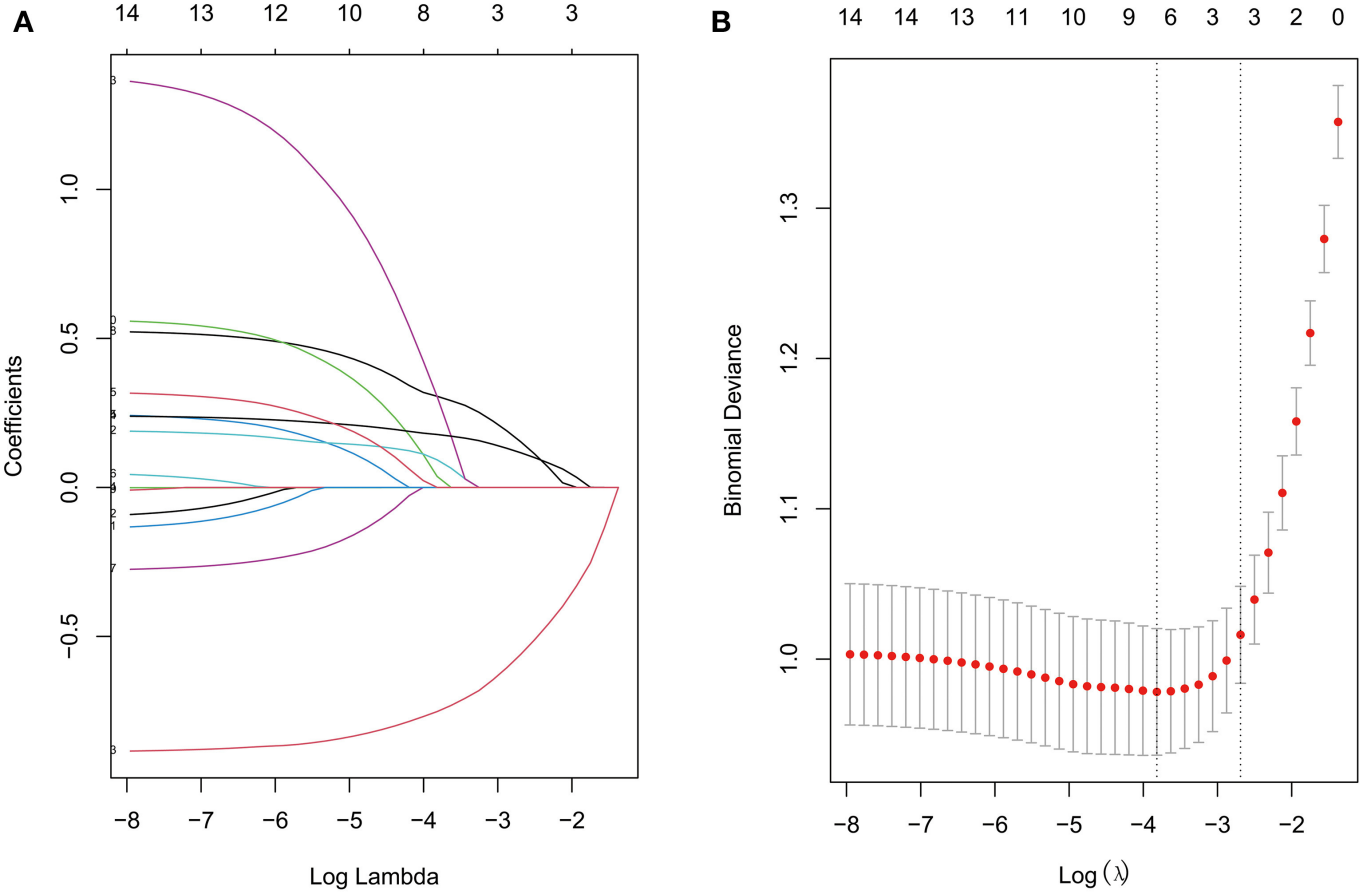
The LASSO regression **(A)** Lasso coefficient profiles of 14 features. **(B)** Feature selection for the predictive model. Turning parameter (λ) selection used 10-fold cross-validation. The vertical axis shows the model misclassification rate, and the horizontal axis shows log(λ). The two vertical dashed lines represent the minimum value and one standard deviation on one side from the minimum value.

**Figure 2:**
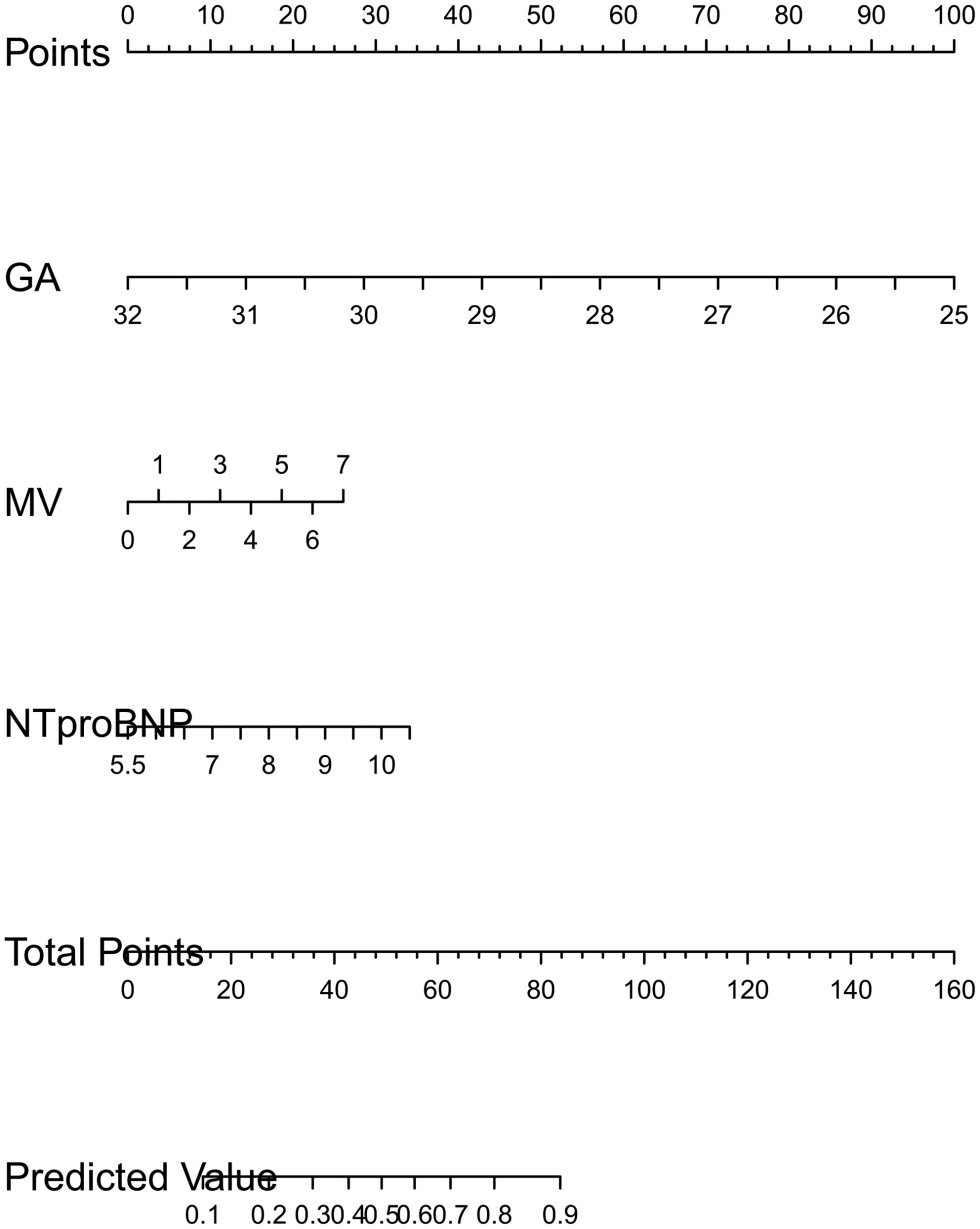
Developed BPD nomogram. GA is gestational age. MV is the time of mechanical ventilation in the 1st week. NTproBNP is the logarithm of NT-pro BNP level based on e. The usage of nomogram. Mark the value of factors on the corresponding axis and draw a vertical line passing the marked point to obtain the corresponding points. Then, we obtain the points together and mark the total points on the “total point” axis. Draw a vertical line passing the marked point and mark where the line crosses the axis of “probability” to read out the probability.

LASSO regression was used to extract the most important predictive factors from a primary dataset. Fourteen variables that might possibly predict BPD were analyzed, and three potential predictors were screened out in the training set. A coefficient profile plot was produced, and a cross-validated error plot of the LASSO regression is shown in Figure 1. The LASSO regression identified GA (OR = 0.40, 95% CI 0.31–0.52), the duration of mechanical ventilation (OR = 1.27, 95% CI 1.12–1.43), and the NT-proBNP level on the 7th day (OR = 1.54, 95% CI 1.17–2.04) as independent predictors of BPD. A predictive nomogram incorporating three factors was developed by logistic regression (Figure 2). The logistic regression equation was

(1) $$\begin{array}{l} \text{lofit}(\text{P})=22.53-0.91\text{ GA}+0.24\text{MV}+0.43\text{ln}(\text{NT}-\text{proBNP}). \end{array}$$

### Validation

**Figure 3:**
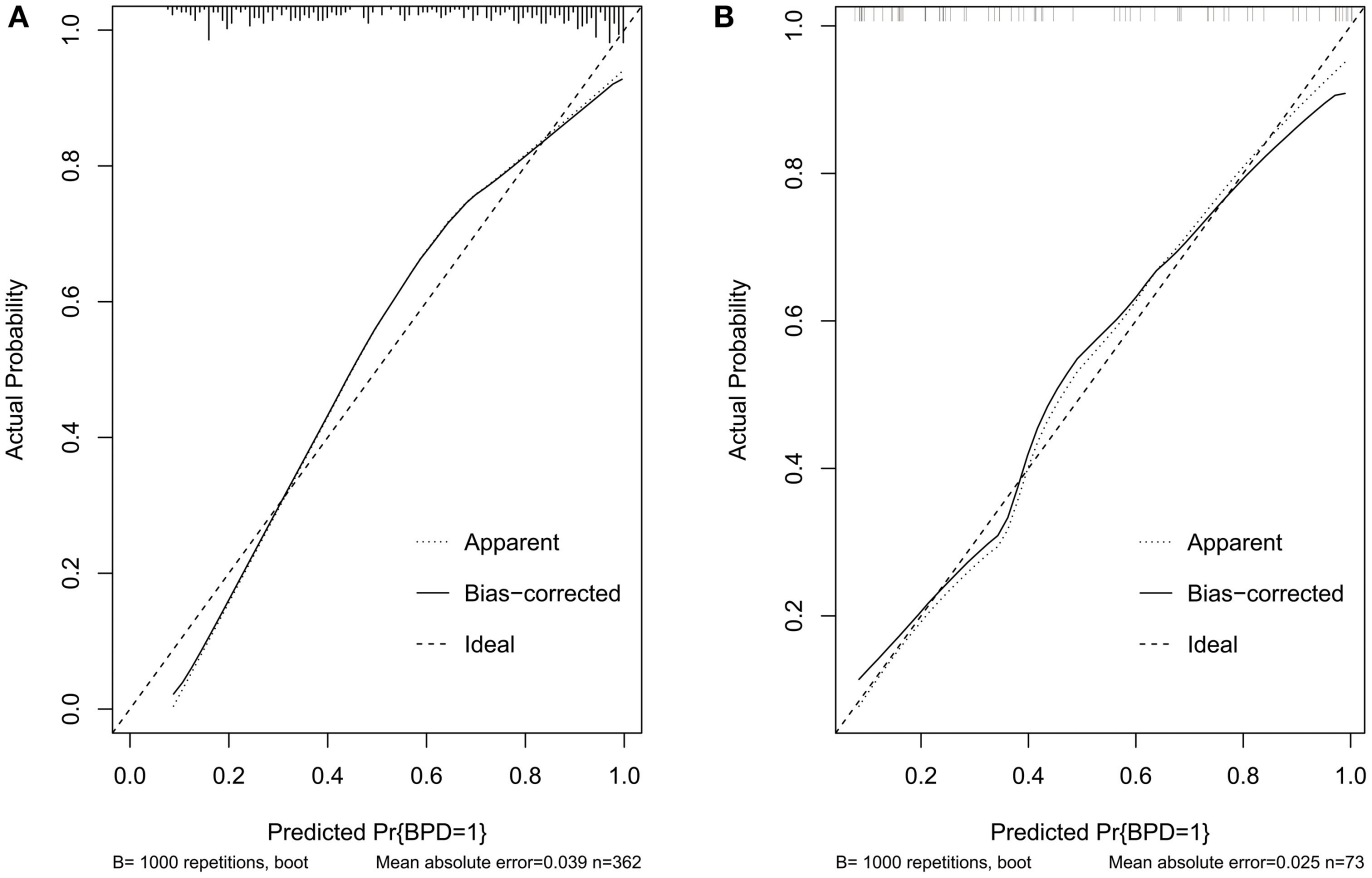
Calibration curves of the BPD prediction model. **(A)** Calibration curves in the training set. **(B)** Calibration curves in the validation set. BPD, bronchopulmonary dysplasia; RDS, neonatal respiratory distress syndrome; CP, cerebral palsy; GA, gestational age; FiO2, fraction of inspiration O2; PMA, postnatal menstrual age; IUGR, intrauterine growth restriction; GDM, gestation diabetes mellitus; PROM, premature rupture of membranes; MODS, multifunctional organ failure; DIC, disseminated intravascular coagulation; NT-proBNP, N-terminal pro-brain natriuretic peptide; RF, respiratory failure; HF, heart failure; PDA, patent ductus arteriosus; AC, fetal abdominal circumference; EFW, estimated fetal weight; UA-AEDF, umbilical artery-absent end-diastolic flow; UtA-PI, uterine artery-pulsatility index; LASSO, least shrinkage and selection operator regression; BNP, B-type natriuretic peptide; PS, pulmonary surfactant; MV, mechanical ventilation; SurE, surfactant without endotracheal tube intubation; NAVA, neurally adjusted ventilatory assist.

Validation of the nomogram was performed with 1,000 bootstrap analyses in the training set. The C-indexes for the prediction model nomogram were 0.853 (95% CI: 0.851–0.854) and 0.855 (95% CI: 0.77–0.94) for the training and validation sets, respectively. The calibration curve (Figure 3) showed good agreement between the nomogram predictions and actual observations in the training set and validation set, and the Hosmer-Lemeshow test showed that there was no significant deviation between the observed and predicted events in the training set (*P* = 0.40) and validation set (*P* = 0.48).

## Discussion

BPD is an important medical condition that causes morbidity and mortality among very-low-birth-weight infants (4). It is affected by multiple factors, including antenatal, postnatal and genetic factors, such as maternal smoking, intrauterine growth restriction, intrauterine inflammation, mechanical ventilation, oxygen supplementation after birth, and cardiovascular malformation (22). In some of these infants, worse cognitive and psychomotor development are also observed later in their development (6). We also considered death to be our outcome measure because death was a competing outcome with BPD (23) and BPD was a major cause of death (4).

In this study, we developed and validated an easy-to-use nomogram as a new approach to diagnose BPD. Fourteen variables were used for construction of the nomogram, which was reduced to three potential predictors using the LASSO regression method. The nomogram incorporated three independent and measurable predictors, including GA, duration of MV and level of NT-proBNP in the 1st week. This nomogram had a good accurate prediction rate of the incidence of BPD or death. The discriminatory ability of this model and the validation results showed that the model performed well. Furthermore, the nomogram may serve as a useful tool for the optimal identification of patients at high risk for BPD or death. Thus, therapeutic decisions will be better informed, and the likelihood of early intervention for high-risk patients will be increased.

This is the first simple-to-use nomogram to predict BPD in Asian areas. Young Don Kim et al. established a scoring method encompassing many respiratory parameters through a logistic regression procedure on different days (24). They found that the discrimination accuracies of the method were 0.76, 0.84, and 0.86 on days 4, 7, and 10, respectively. Li Ding et al. built a model involving neonatal critical illness score, enteral feeding ≥40 days after birth and other factors (25). The ROC curve showed a high AUC value of 0,96. Our study has a median but similar AUC of 0.85. Compared with the other models mentioned above, we could calculate the risk of BPD earlier and have a better discrimination, and we also had other advantages, such as using LASSO regression for analyzing clinical factors. The biggest difference is that our study used NT-proBNP to estimate the risk.

NT-proBNP was included in the model as a predictor. Over the years, there has been increasing recognition that new biochemical markers, such as NT-proBNP, are useful in the diagnosis of BPD (26). Pro BNP synthesized and secreted by myocardial cells is cleaved into bioactive B-type natriuretic peptide (BNP) and inactive NT-proBNP when volume and pressure are overloaded. Studies have demonstrated that serum NT-proBNP appears to be a good screening tool in BPD-associated pulmonary hypertension (12), and Blanco et.al demonstrated the NT pro-BNP of 5–10 days after birth may identify preterm infants with high risk of BPD (26). In our study, preterm infants in the BPD group displayed significantly higher levels of NT-proBNP in the serum than those in the non-BPD group. After adjusting for the other factors, the odds ratio was 1.54 (95% CI 1.17–2.04). Our previous studies showed that NT-proBNP can be used to predict weaning failure for premature patients with RDS, and a high level of NT-proBNP corresponded with a high failure rate of extubation (27), which would increase the risk of BPD (28). Thus, the early use of NT-proBNP could identify high-risk patients contributing to reducing the risk of BPD. In an animal model, anti-sFlt-1 (an antibody to an endogenous antagonist of vascular endothelial growth factor) treatment preserved lung structure and function (29). A clinical study found that NT-proBNP might contribute to inhibiting angiogenesis by increasing sVEGFR-1, which is a VEGF inhibitor (30). These studies suggested that NT-proBNP might participate in the pathogenesis of BPD. Therefore, monitoring serum NT-proBNP levels may aid in the early prediction of BPD development.

In addition, some recent studies have shown that urinary NT-proBNP might be a new biomarker for BPD in neonates. NT-proBNP was removed from plasma via passive excretion by organs with high blood flow, including the kidney (31). The examination of urinary NT-proBNP required less cost and non-invasive measurement, suggesting that it might be a useful potential marker. Urinary NT-proBNP was proven to be elevated at 7, 14, and 28 days of life in infants with ROP or BPD (32). In a retrospective study containing 54 premature babies, urinary NT-proBNP levels were higher in the BPD-PH group than in the BPD and control groups, and a cutoff level of 2,345 pg/mL at 28 weeks of GA had a sensitivity and specificity of 83.3 and 84.2%, respectively (33). Since there were several limitations due to the lack of research and the small sample size, the results need to be validated in further data. Considering the above, we incorporated serum NT-proBNP instead of urinary NT-proBNP into the nomogram.

Prematurity was a key factor in infants with BPD (OR = 0.40, 95% CI 0.31–0.52). It was a critical window of rapid lung development in the last 3 months; therefore, premature infants lacked the normal progress of alveolarization and angiogenesis (34). Because they must use their developing lungs for survival, they are especially fragile to damage, contributing to the pathogenesis of BPD. Prematurity would also impaired lung function. Hjalmarson et al. studied 32 healthy preterm infants and 53 healthy full-term infants and found that lung function was decreased in preterm infants (35). They also demonstrated that specific compliance and conductance decreased in prematurity, suggesting that preterm birth *per se* affected alveolarization and the formation of elastic tissue in the lungs.

The finding that mechanical ventilation is a predictive factor is consistent with the existing literature. MV is undoubtedly one of the key advances in neonatal care, even in this non-invasive respiratory era. Most extremely low birth weight infants received invasive ventilation on the 1st day of life (36). Mechanical ventilation has many untoward effects, although it is often lifesaving. Ventilation breaks the blood-gas barrier, leaking plasma-derived fluid and proteins into airspaces and interfering with the function of pulmonary surfactant in the remaining open area (37). 15 min of a high tidal volume was demonstrated to induce inflammatory cytokine expression (38) in animal models, and inflammation was a key contributor to the pathogenesis of BPD (39). In addition, mechanical injury and hyperoxia caused by MV could affect normal alveolar development, which leads to the pathogenesis of BPD. In theory, non-invasive ventilation offers the benefit of avoiding the use of an endotracheal tube and avoiding the contribution of the postnatal inflammatory response to the development of BPD. Although an early cohort compared CPAP with MV, suggesting a large reduction in the risk of BPD, multiple recent studies showed a much more modest benefit of avoiding MV (40). Therefore, we should develop individual ventilation strategies to address each patient's condition rather than blindly avoiding them. Moreover, new ventilation strategies were found to be applied to BPD prevention. The application of neurally adjusted ventilatory assist ventilation (NAVA) in newborns with BPD has rarely been reported. Some studies have proven that NAVA could be safely used in BPD patients and decrease the need for sedation (41).

There were still several limitations in our study. Our data were collected in a single center and might not be representative of all BPD patients. In a state collaborative retrospective cohort study, BPD occurrence varied widely across different hospitals (42). Multicenter cohort and external validation should be performed. As the data were collected retrospectively, no causal relationship can be determined from the predictive factors on the observed outcomes. We excluded inherited metabolic disorders and multiple malformations; therefore, this study is not generalizable to those specific groups. In addition, our incidence of BPD was 52.6% higher than the incidence in Europe (43). Beyond ethnicity, preterm newborns under 28 weeks had a lower survival rate (44), but infants in our study cohort were treated for more than 1 week.

In conclusion, we developed a nomogram with good accuracy to help clinicians assess the risk of BPD at 7 days of life. An estimate of individual risk would help clinicians recognize high-risk patients and implement timely interventions. Furthermore, these predictors provide new ideas for BPD treatment, such as non-invasive ventilation and new drugs requiring further study. We will also extend the usage of the nomogram in BPD in further prospective studies.

## Data Availability Statement

The original contributions generated for this study are included in the article/Supplementary Material, further inquiries can be directed to the corresponding author/s.

## Ethics Statement

The studies involving human participants were reviewed and approved by the ethics committee of the First Affiliated Hospital of Zhengzhou University. Written informed consent to participate in this study was provided by the participants' legal guardian/next of kin.

## Author Contributions

QZ conceptualized and designed the study. All the authors contributed ideas to help consummate the study. JdZ and CL drafted the initial manuscript. MS organized the database. ML, XC, ZS, YZ, LlW, and LiW carried out the date analyses and helped to explain the outcomes. LiW, FM, and JuZ made modifications and additions to the manuscript. SZ, QX, SH, and MZ made modifications and data collections to the revised manuscript. All authors contributed to manuscript revision, read and approved the submitted version.

## Conflict of Interest

The authors declare that the research was conducted in the absence of any commercial or financial relationships that could be construed as a potential conflict of interest.

## References

[1] Northway WH Jr Rosan RC Porter DY. Pulmonary disease following respirator therapy of hyaline-membrane disease. Bronchopulmonary dysplasia. N Engl J Med. (1967) 276:357–68 10.1056/NEJM196702162760701 5334613

[2] Gortner L Misselwitz B Milligan D Zeitlin J Kollee L Boerch K. Rates of bronchopulmonary dysplasia in very preterm neonates in Europe: results from the MOSAIC cohort. Neonatology. (2011) 99:112–7. 10.1159/000313024 20733331

[3] Stoll BJ Hansen NI Bell EF Walsh MC Carlo WA Shankaran S. Trends in Care Practices, Morbidity, and Mortality of Extremely Preterm Neonates, 1993-2012. JAMA. (2015) 314:1039–51. 10.1097/01.aoa.0000482610.95044.1b 26348753 PMC4787615

[4] Zhou J Ba Y Du Y Lin SB Chen C Chinese Collaborative Study Group for Etiologies of NICU Deaths. The etiology of neonatal intensive care unit death in extremely low birth weight infants: a multicenter survey in China. Am J Perinatol. (2020). 10.1055/s-0040-1701611. [Epub ahead of print]. 32102093

[5] Baraldi E Filippone M. Chronic lung disease after premature birth. N Engl J Med. (2007) 357:1946–55. 10.1056/NEJMra067279 17989387

[6] Gou X Yang L Pan L Xiao D. Association between bronchopulmonary dysplasia and cerebral palsy in children: a meta-analysis. BMJ Open. (2018) 8:e020735. 10.1136/bmjopen-2017-020735 30232102 PMC6150141

[7] Goeman JJ. L1 penalized estimation in the Cox proportional hazards model. Biom J. (2010) 52:70–84. 10.1002/bimj.200900028 19937997

[8] Liu R Yuan M Xu H Chen P Xu XS Yang Y. Adaptive weighted sum tests via LASSO method in multi-locus family-based association analysis. Comput Biol Chem. (2020) 88:107320. 10.1016/j.compbiolchem.2020.107320 32711355

[9] Onland W Debray T Laughon M Miedema M Cools F Askie L. Clinical prediction models for bronchopulmonary dysplasia: a systematic review and external validation study. BMC Pediatr. (2013)13:207. 10.1186/1471-2431-13-207 24345305 PMC3878731

[10] Cohen A Taeusch HW. Prediction of risk of bronchopulmonary dysplasia. Am J Perinatol. (1983) 1:21–2. 10.1055/s-2007-1000045 6680646

[11] Laughon MM Langer JC Bose CL Smith PB Ambalavanan N Kennedy KA. Prediction of bronchopulmonary dysplasia by postnatal age in extremely premature infants. Am J Respir Crit Care Med. (2011) 183:1715–22. 10.1164/rccm.201101-0055OC 21471086 PMC3136997

[12] Dasgupta S Aly AM Malloy MH Okorodudu AO Jain SK. NTproBNP as a surrogate biomarker for early screening of pulmonary hypertension in preterm infants with bronchopulmonary dysplasia. J Perinatol. (2018) 38:1252–7. 10.1038/s41372-018-0164-1 29977013

[13] Jobe AH Bancalari E. Bronchopulmonary dysplasia. Am J Respir Crit Care Med. (2001) 163:1723–9. 10.1164/ajrccm.163.7.2011060 11401896

[14] Gordijn S Beune I Thilaganathan B Papageorghiou A Baschat A Baker P. Consensus definition of fetal growth restriction: a Delphi procedure. Ultrasound Obstet Gynecol. (2016) 48:333–9. 10.1002/uog.15884 26909664

[15] Girsen AI Wallenstein MB Davis AS Hintz SR Desai AK Mansour T. Effect of antepartum meconium staining on perinatal and neonatal outcomes among pregnancies with gastroschisis. J Matern Fetal Neonatal Med. (2016) 29:2500–4. 10.3109/14767058.2015.1090971 26445130

[16] Elimian A Lawlor P Figueroa R Wiencek V Garry D Quirk JG. Intrapartum assessment of fetal well-being: any role for a fetal admission test? J Matern Fetal Neonatal Med. (2003) 13:408–13. 10.1080/jmf.13.6.408.413 12962267

[17] ACOG Practice Bulletin No. 202: gestational hypertension and preeclampsia. Obstet Gynecol. (2019) 133:1. 10.1097/AOG.0000000000003018 30575675

[18] ACOG Practice Bulletin No. 190: gestational diabetes mellitus. Obstet Gynecol. (2018) 131:e49–64. 10.1097/AOG.0000000000002501 29370047

[19] Canavan T Simhan HN Caritis S. An evidence-based approach to the evaluation and treatment of premature rupture of membranes: Part I. Obstet Gynecol Surv. (2004) 59:669–77. 10.1097/01.ogx.0000137610.33201.a4 15329560

[20] Powell MB Ahlers-Schmidt CR Engel M Bloom BT. Clinically significant cardiopulmonary events and the effect of definition standardization on apnea of prematurity management. J Perinatol. (2017) 37:88–90. 10.1038/jp.2016.167 27684421

[21] Sweet DG Carnielli V Greisen G Hallman M Ozek E Plavka R. European Consensus Guidelines on the Management of Respiratory Distress Syndrome - 2016 Update. Neonatology. (2017) 111:107–25. 10.1159/000448985 27649091

[22] Álvarez-Fuente M Moreno L Mitchell J Reiss I Lopez P Elorza D. Preventing bronchopulmonary dysplasia: new tools for an old challenge. Pediatr Res. (2019) 85:432–41. 10.1038/s41390-018-0228-0 30464331

[23] Andersen PK Geskus RB de Witte T Putter H. Competing risks in epidemiology: possibilities and pitfalls. Int J Epidemiol. (2012) 41:861–70. 10.1093/ije/dyr213 22253319 PMC3396320

[24] Kim Y Kim E Kim K Pi S Kang W. Scoring method for early prediction of neonatal chronic lung disease using modified respiratory parameters. J Korean Med Sci. (2005) 20:397–401. 10.3346/jkms.2005.20.3.397 15953859 PMC2782193

[25] Ding L Wang H Geng H Cui N Huang F Zhu X. Prediction of bronchopulmonary dysplasia in preterm infants using postnatal risk factors. Front Pediatr. (2020) 8:349. 10.3389/fped.2020.00349 32676490 PMC7333538

[26] Rodríguez-Blanco S Oulego-Erroz I Alonso-Quintela P Terroba-Seara S Jiménez-González A Palau-Benavides M. N-terminal-probrain natriuretic peptide as a biomarker of moderate to severe bronchopulmonary dysplasia in preterm infants: a prospective observational study. Pediatr Pulmonol. (2018) 53:1073–81. 10.1002/ppul.24053 29790673

[27] Zhang Q Shi Z Luo C Wang L Zhang S Cheng X. Application of NT-proBNP in ventilator weaning for preterm infants with RDS. Pediatr Pulmonol. (2014) 49:757–63. 10.1002/ppul.22875 24019216

[28] Shalish W Kanbar L Kovacs L Chawla S Keszler M Rao S. The impact of time interval between extubation and reintubation on death or bronchopulmonary dysplasia in extremely preterm infants. J Pediatr. (2019) 205:70–76.e2. 10.1016/j.jpeds.2018.09.062 30404739

[29] Wallace B Peisl A Seedorf G Nowlin T Kim C Bosco J. Anti-sFlt-1 therapy preserves lung alveolar and vascular growth in antenatal models of bronchopulmonary dysplasia. Am J Respir Crit Care Med. (2018) 197:776–87. 10.1164/rccm.201707-1371OC 29268623 PMC5855071

[30] Failla CM Carbo M Morea V. Positive and negative regulation of angiogenesis by soluble vascular endothelial growth factor Receptor-1. Int J Mol Sci. (2018) 19:1306. 10.3390/ijms19051306 29702562 PMC5983705

[31] Mair J. Biochemistry of B-type natriuretic peptide–where are we now? Clin Chem Lab Med. (2008) 46:1507–14. 10.1515/CCLM.2008.295 18842106

[32] Czernik C Metze B Müller C Müller B Bührer C. Urinary N-terminal B-type natriuretic peptide predicts severe retinopathy of prematurity. Pediatrics. 128 (2011) e545–9. 10.1542/peds.2011-0603 21824875

[33] Naeem B Ayub A Aly AM Malloy MH Okorodudu AO Jain SK. Urinary NT-proBNP as a potential noninvasive biomarker for screening of pulmonary hypertension in preterm infants: a pilot study. J Perinatol. (2020) 40:628–32. 10.1038/s41372-019-0581-9 31911650

[34] Thébaud B Goss KN Laughon M Whitsett JA Abman SH Steinhorn RH. Bronchopulmonary dysplasia. Nat Rev Dis Prim. (2019) 5:78. 10.1038/s41572-019-0127-7 31727986 PMC6986462

[35] Hjalmarson O Sandberg K. Abnormal lung function in healthy preterm infants. Am J Respir Crit Care Med. (2002) 165:83–7. 10.1164/ajrccm.165.1.2107093 11779735

[36] Walsh M Morris B Wrage L Vohr B Poole W Tyson J. Extremely low birthweight neonates with protracted ventilation: mortality and 18-month neurodevelopmental outcomes. J Pediatr. (2005) 146:798–804. 10.1016/j.jpeds.2005.01.047 15973322

[37] Bates J Smith BJ. Ventilator-induced lung injury and lung mechanics. Ann Transl Med. (2018) 6:378. 10.21037/atm.2018.06.29 30460252 PMC6212358

[38] Hillman NH Polglase GR Pillow JJ Saito M Kallapur SG Jobe AH. Inflammation and lung maturation from stretch injury in preterm fetal sheep. Am J Physiol Lung Cell Mol Physiol. (2011) 300:L232–41. 10.1152/ajplung.00294.2010 21131401 PMC3043810

[39] Coalson JJ. Pathology of new bronchopulmonary dysplasia. Semin Neonatol.. (2003) 8:73–81. 10.1016/S1084-2756(02)00193-8 12667832

[40] Keszler M Sant'Anna G. Mechanical Ventilation and Bronchopulmonary Dysplasia. Clin Perinatol. (2015) 42:781–96. 10.1016/j.clp.2015.08.006 26593078

[41] Rong X Liang F Li Y-J Liang H Zhao X-P Zou H-M. Application of Neurally Adjusted Ventilatory Assist in Premature Neonates Less Than 1,500 Grams With Established or Evolving Bronchopulmonary Dysplasia. Front Pediatr. (2020) 8:110. 10.3389/fped.2020.00110 32266188 PMC7105827

[42] Lapcharoensap W Gage SC Kan P Profit J Shaw GM Gould JB. Hospital variation and risk factors for bronchopulmonary dysplasia in a population-based cohort. JAMA Pediatr. (2015) 169:e143676. 10.1001/jamapediatrics.2014.3676 25642906

[43] Rutkowska M Hozejowski R Helwich E Borszewska-Kornacka M Gadzinowski J. Severe bronchopulmonary dysplasia - incidence and predictive factors in a prospective, multicenter study in very preterm infants with respiratory distress syndrome. J Matern Fetal Neonatal Med. (2019) 32:1958–64. 10.1080/14767058.2017.1422711 29295665

[44] Kroll M Kurinczuk J Hollowell J Macfarlane A Li Y Quigley MA. Ethnic and socioeconomic variation in cause-specific preterm infant mortality by gestational age at birth: national cohort study. BMJ J. (2020) 105:56–63. 10.1136/archdischild-2018-316463 31123058 PMC6951229

